# Inhibition of LINE-1 Retrotransposition by Capsaicin

**DOI:** 10.3390/ijms19103243

**Published:** 2018-10-19

**Authors:** Yuki Nishikawa, Ryota Nakayama, Shunsuke Obika, Eriko Ohsaki, Keiji Ueda, Tomoyuki Honda

**Affiliations:** Division of Virology, Department of Microbiology and Immunology, Osaka University Graduate School of Medicine, Osaka 565-0871, Japan; u548524d@ecs.osaka-u.ac.jp (Y.N.); rnakayama@virus.med.osaka-u.ac.jp (R.N.); u687305i@ecs.osaka-u.ac.jp (S.O.); eohsaki@virus.med.osaka-u.ac.jp (E.O.); kueda@virus.med.osaka-u.ac.jp (K.U.)

**Keywords:** anti-cancer, capsaicin, inhibitor, LINE-1, L1, phytochemicals, retrotransposition, reverse transcriptase

## Abstract

Long interspersed nuclear element 1 (LINE-1 or L1) is a non-long terminal repeat (LTR) retrotransposon that constitutes approximately 17% of the human genome. Since approximately 100 copies are still competent for retrotransposition to other genomic loci, dysregulated retrotransposition of L1 is considered to be a major risk factor of endogenous mutagenesis in humans. Thus, it is important to find drugs to regulate this process. Although various chemicals are reportedly capable of affecting L1 retrotransposition, it is poorly understood whether phytochemicals modulate L1 retrotransposition. Here, we screened a library of compounds that were derived from phytochemicals for reverse transcriptase (RT) inhibition with an in vitro RT assay. We identified capsaicin as a novel RT inhibitor that also suppressed L1 retrotransposition. The inhibitory effect of capsaicin on L1 retrotransposition was mediated neither through its receptor, nor through its modulation of the L1 promoter and/or antisense promoter activity, excluding the possibility that capsaicin indirectly affected L1 retrotransposition. Collectively, capsaicin suppressed L1 retrotransposition most likely by inhibiting the RT activity of L1 ORF2p, which is the L1-encoded RT responsible for L1 retrotransposition. Given that L1-mediated mutagenesis can cause tumorigenesis, our findings suggest the potential of capsaicin for suppressing cancer development.

## 1. Introduction

The eukaryotic genome contains retrotransposable elements, also known as retrotransposons, with or without long terminal repeats (LTRs). Long interspersed nuclear element 1 (LINE-1 or L1) is a non-LTR retrotransposon and constitutes approximately 17% of the human genome [1]. L1 consists of a 5′ untranslated region (UTR), two open reading frames (ORFs) that encode two proteins, ORF1p and ORF2p, and a 3′ UTR with a polyadenylation signal. ORF1p is an RNA-binding protein with nucleic acid chaperone activity that is required for L1 retrotransposition [2]. ORF2p is a protein with endonuclease and reverse transcriptase (RT) activity responsible for a “copy-and-paste” retrotransposition of L1s to new genomic loci [3,4]. Although most L1s are truncated and therefore defective for retrotransposition activity, approximately 100 copies are still competent [3,4].

The active retrotransposition of L1 is considered to be a major source of endogenous mutagenesis in humans [5,6]. In addition, de novo L1 insertions can affect gene expression [7,8]. As a result of these effects, L1 retrotransposition may potentially result in the development of human diseases. For example, dysregulation of L1 has been reported in various diseases such as cancers and schizophrenia [9,10,11]. Natural L1 insertions into genes encoding for factor VIII and dystrophin have been found in hemophilia A and muscular dystrophy cases, respectively [12,13]. Thus, it is important to find drugs that modulate L1 retrotransposition activity. Environmental factors, such as chemicals, oxidative stress, and infection, are reportedly capable of affecting L1 retrotransposition [9,11,14,15]. Although several chemicals have been reported to affect L1 retrotransposition [14], knowledge regarding phytochemicals that may modulate L1 retrotransposition is still lacking. 

Capsaicin is a major chemical constituent of *Asiasari Radix* and *Capsicum annuum* [16] and also a constituent of a Chinese traditional herbal medicine, *Sho-seiryu-to*. The compound is a homovanillic acid derivative and is known as a potent analgesic that is used for treating pain and inflammation [17]. The analgesic effect of capsaicin is mediated through its interaction with the transient receptor potential vanilloid member 1 (TRPV1) [18]. However, the effect of capsaicin on L1 retrotransposition has not yet been studied. 

Here, we screened a library of compounds that were derived from natural medicines for RT inhibition by an in vitro RT assay with Moloney murine leukemia virus (M-MLV) RT. Among the 96 compounds we examined, we found that capsaicin inhibited the activity of M-MLV RT. Capsaicin also suppressed L1 retrotransposition in a dose-dependent manner, while it did not affect the L1 promoter and antisense promoter activities. These results suggest the potential of capsaicin as a novel RT inhibitor that can inhibit L1 retrotransposition. 

## 2. Results

### 2.1. Capsaicin Inhibits Activity of Retroviral RT 

**Figure 1:**
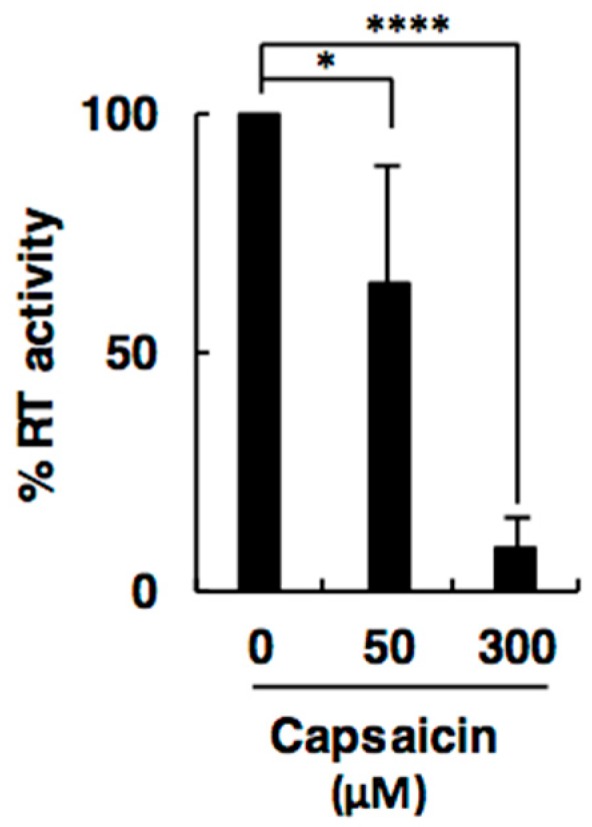
Capsaicin inhibits the activity of a retroviral reverse transcriptase (RT). The effect of capsaicin on activity of Moloney murine leukemia virus (M-MLV) RT was evaluated using an in vitro RT assay. Values are expressed as the means + S.E. of four independent experiments. * *p* < 0.05; **** *p* < 0.001.

To identify novel RT inhibitors that can affect L1 retrotransposition, we sought to screen compounds that inhibit RT activity. For efficient screening, we used M-MLV RT as a model RT, because M-MLV RT shares several features with L1 ORF2p [19] and was readily commercially available. We screened 96 compounds that were derived from natural medicines in the Institute of Natural Medicine (INM) deposited WAKANYAKU library (Institute of Natural Medicine, University of Toyama, Table S1) to test the effects on M-MLV RT at a final concentration of 300 µM using an in vitro M-MLV RT assay. Among these compounds, we found that capsaicin reproducibly inhibited the activity of M-MLV RT in a dose-dependent manner (Figure 1). 

### 2.2. Capsaicin Suppresses L1 Retrotransposition 

**Figure 2:**
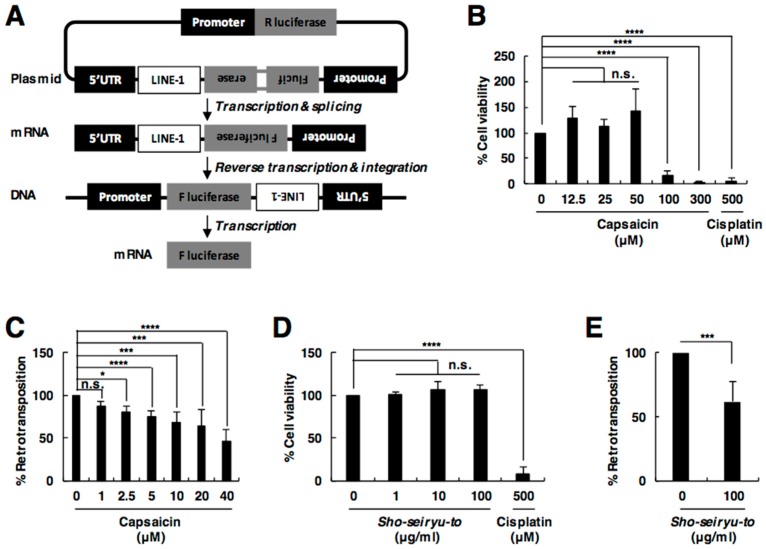
Capsaicin suppresses L1 retrotransposition. (**A**) The rationale for L1 retrotransposition assay. L1 with a retrotransposition reporter cassette is expressed under the L1 cognate promoter that is located in the 5′ UTR of pYX014. The L1 retrotransposition reporter is the *Firefly luciferase* (*F. luciferase)* gene interrupted by an antisense intron, which has its own promoter (Pro), and is expressed from the antisense strand relative to the L1 promoter. Only after L1 transcription, splicing, reverse transcription of the spliced L1 mRNA, and integration into the host genome, the *Firefly* luciferase activity is detected. Activity of the *Renilla luciferase* (*R. luciferase*) gene is measured as a transfection control. (**B**,**D**) Effect of capsaicin (**B**) or *Sho-seiryu-to* (**D**) on cell viability. 293T cells were incubated with capsaicin (**B**) or *Sho-seiryu-to* (**D**) at the indicated concentrations for 3 days. (**C**,**E**) The effect of capsaicin (**C**) or *Sho-seiryu-to* (**E**) on L1 retrotransposition. 293T cells were transfected with the L1 retrotransposition reporter construct. Capsaicin (**C**) or Sho-seiryu-to (**E**) was added at the indicated concentrations at 24 h post-transfection. Luciferase activity in the cells was evaluated at 4 days post-transfection. Values are expressed as the means + S.E. of at least three independent experiments. * *p* < 0.05; *** *p* < 0.005; **** *p* < 0.001; n.s., no significance.

To evaluate the effect of capsaicin on L1, we examined L1 retrotransposition in the presence of capsaicin using an established dual-luciferase-based L1 retrotransposition assay (Figure 2A) [20]. We first examined the cytotoxic effect of capsaicin to determine the test range. Cisplatin, which is an apoptosis-inducing anti-cancer drug [21], exhibited cytotoxicity (Figure 2B,D). On the other hand, capsaicin did not show any cytotoxicity at concentrations of 2.5–50 µM, while it did at concentrations higher than 100 µM (Figure 2B). Therefore, we decided to use capsaicin at concentrations lower than 50 µM for further study. The dual-luciferase-based L1 retrotransposition assay showed that capsaicin inhibited L1 retrotransposition in a dose-dependent manner and IC_50_ was calculated as 34.6 µM (Figure 2C). Furthermore, *Sho-seiryu-to*, which contains capsaicin, also suppressed L1 retrotransposition without any cell toxicity (Figure 2D,E). These results indicate that capsaicin, a novel inhibitor of an RT, also inhibits L1 retrotransposition. 

### 2.3. The Effect of Capsaicin on L1 Is Independent of TRPV1 

**Figure 3:**
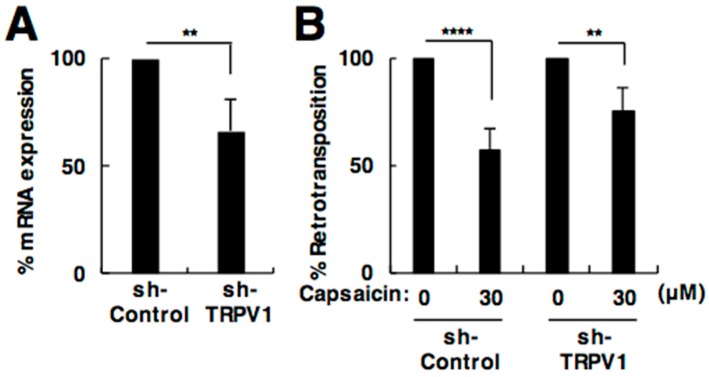
The effect of capsaicin on L1 is independent of TRPV1. (**A**) Expression of TRPV1 by transfection of plasmids expressing sh-TRPV1. (**B**) Effect of capsaicin on L1 retrotransposition in TRPV1 knockdown cells. 293T cells were transfected with the L1 retrotransposition reporter construct, together with a plasmid expressing sh-TRPV1. After 24 h, 30 µM capsaicin was added. Luciferase activity in the cells was evaluated at 4 days post-transfection. Values are expressed as the means + S.E. of at least four independent experiments. ** *p* < 0.01; **** *p* < 0.001.

To exclude the possibility that capsaicin indirectly regulated L1 retrotransposition through its receptor binding and signaling cascade, we knocked down TRPV1, a capsaicin endogenous receptor [18], and evaluated the inhibitory effect of capsaicin on L1. Co-transfection of a plasmid that expressed shRNA against TRPV1 with the L1 retrotransposition reporter construct knocked down the expression of TRPV1 (Figure 3A). In the TRPV1 knockdown condition, capsaicin still suppressed L1 retrotransposition (Figure 3B). These results suggest that capsaicin likely modulates L1 retrotransposition not through receptor binding.

### 2.4. Capsaicin Does Not Affect L1 Promoter or Antisense Promoter Activity 

**Figure 4:**
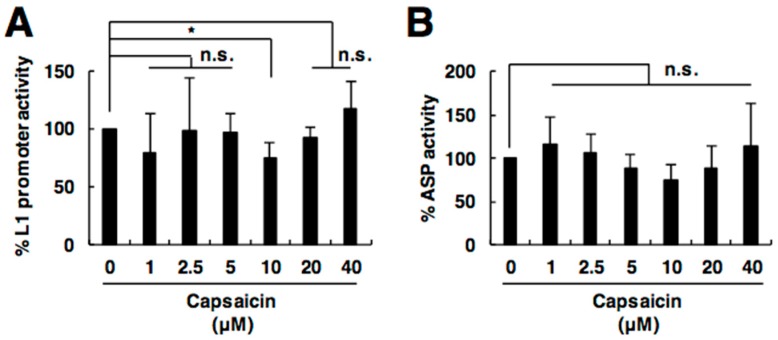
Capsaicin does not affect L1 promoter or antisense promoter (ASP) activity. (**A**) Effect of capsaicin on L1 5′ UTR promoter activity. (**B**) Effect of capsaicin on L1 ASP activity. 293T cells were transfected with pGLuc-5′-UTR (**A**) or pGLuc-ASP (**B**), together with pCMV-CLuc. Capsaicin was added at the indicated concentrations at 24 h post-transfection. Luciferase activity in the cells was evaluated at 4 days post-transfection. Values are expressed as the means + S.E. of at least three independent experiments. * *p* < 0.05; n.s., no significance.

To reveal the mechanism of how capsaicin inhibits L1 retrotransposition, we next evaluated its effect on L1 expression. Since the L1 5′ UTR promoter activity is responsible for L1 RNA expression, we conducted an L1 5′ UTR promoter assay. We detected a slight reduction in the L1 5′ UTR promoter activity by capsaicin only at a concentration of 10 µM, but did not detect any significant dose-dependent suppression of promoter activity in the range of its L1 inhibitory effect (Figure 4A). Recently, the primate L1 5′ UTR was reported to contain a primate-specific ORF in antisense orientation, *ORF0*, whose expression enhances L1 retrotransposition [22]. Therefore, we hypothesized that capsaicin reduces the expression of ORF0 and thereby suppresses L1 retrotransposition. Since the expression of ORF0 is regulated by the L1 antisense promoter, located in the 5′ UTR in antisense orientation, we evaluated the activity of the L1 antisense promoter (ASP). However, capsaicin did not affect the activity of L1 ASP in the test range (Figure 4B). These results indicate that capsaicin does not regulate L1 retrotransposition via either transcriptional control of L1 itself or *ORF0*. Taken together, it is likely that capsaicin suppresses L1 retrotransposition through its direct modulation of components of the L1 ribonucleoprotein complex (RNP). 

## 3. Discussion

Phytochemicals are beneficial for therapeutic use because they are active in many biological processes and have less toxicity than pharmaceutical agents. In this report, we tested the effects of such chemicals on L1 retrotransposition. We identified capsaicin as a novel RT inhibitor that suppresses L1 retrotransposition. Since capsaicin suppressed the activity of M-MLV RT, did not show dose-dependent suppression of the L1 promoter and antisense promoter activities, and was still active in the TRPV1-knocked down condition, it is likely that capsaicin suppressed L1 by inhibiting the RT activity of L1 ORF2p. 

A main concern of this study is the use of an in vitro RT assay with M-MLV RT to screen compounds that regulate L1 retrotransposition. Indeed, L1 ORF2p, the RT of L1, was more suitable for our purpose, but the use of M-MLV RT provided several advantages over the use of L1 ORF2p. First, quality-certified M-MLV RT was commercially available from Thermo Fisher Scientific and ready to use for screening. Second, the reaction condition of the in vitro M-MLV RT assay has been already established in the kit, making it easy to set up screening. Third, some nucleoside RT inhibitors of retroviral RT are also known to inhibit L1 RT, which suggests that M-MLV RT and L1 ORF2p share several properties [19]. Additionally, by using M-MLV RT for this screening, we expected to get inhibitors that act on a broad range of RTs. In this study, we demonstrated the inhibitory effect of capsaicin only on L1 retrotransposition in vivo. Considering that endogenous retroviruses (retrotransposons with LTRs) are derived from ancient retrovirus infections [23] and that we demonstrated the inhibitory effect of capsaicin on the activity of a retroviral RT in vitro, we speculate that capsaicin can broadly inhibit retrotransposition mediated by L1 and other types of endogenous retroviruses. Furthermore, by using M-MLV RT for this screening, we could speculate the sites of L1 ORF2p that were targeted by capsaicin; it is unlikely that capsaicin targeted the “non-nucleoside RT inhibitor pocket” of RTs, because capsaicin inhibited both the retroviral and L1 RTs, yet sequence alignment reveals the difference in the pocket of these RTs [19]. 

Emerging evidence indicates that capsaicin exhibits an anti-cancer and/or growth-inhibitory effect in various cancers [16,24]. Although the mechanisms of how capsaicin exhibits these inhibitory effects against various cancer cell types still remain unclear, the proposed mechanisms are related to cell cycle arrest and induction of apoptosis [25]. Capsaicin seems to induce apoptosis via intracellular calcium increase, reactive oxygen species, and disruption of the mitochondrial membrane potential [24]. The pro-apoptotic activity of capsaicin is mediated through TRPV1 in many cancers [26]. The p53 protein, which regulates cell cycle arrest, DNA repair, and apoptosis, is proposed to be a target of the anti-cancer activity of capsaicin because capsaicin upregulates p53 and thereby induces the expression of pro-apoptotic genes [27]. Capsaicin also inhibits cancer cell proliferation by cell cycle arrest through the inhibition of cyclin-dependent kinases [28]. In this study, we found a novel activity of capsaicin, the inhibition of L1 retrotransposition. Since L1 retrotransposition is proposed to be mutagenic and can cause tumorigenesis [9,10], capsaicin may suppress the progression of tumorigenesis through the inhibition of L1-mediated mutagenesis. Collectively, capsaicin possesses several properties that exhibit anti-cancer activities and can therefore be a potent candidate for use in anti-cancer therapies, especially in the case of certain cancers where L1 plays a role in tumorigenesis [9,10]. 

In conclusion, we demonstrated the potential of capsaicin for inhibiting retroviral RT activity and suppressing L1 retrotransposition, possibly via inhibition of RT activity of ORF2p. Our findings suggest the potential of capsaicin for suppressing cancer development and they may facilitate the studies of capsaicin or related compounds, capsaicinoids, for cancer prevention and treatment. 

## 4. Materials and Methods 

### 4.1. Cells

293T cells (a human embryonic kidney cell line) were cultured in Dulbecco’s modified Eagle’s medium (DMEM, Nacalai, Kyoto, Japan) supplemented with 5% fetal bovine serum (FBS). 

### 4.2. Chemicals

The compound library that was derived from natural medicines, the INM deposited WAKANYAKU library, was kindly supplied from the Institute of Natural Medicine, University of Toyama. Capsaicin was purchased from Sigma-Aldrich (St. Louis, MO, USA). The *Sho-seiryu-to* extract was included in the INM deposited WAKANYAKU library. 

### 4.3. Plasmids

The reporter plasmid for L1 retrotransposition, pYX014, was kindly provided by Dr. Wenfeng An (South Dakota State University, USA) [20]. The reporter plasmid for L1 promoter activity, pGLuc-5′-UTR, was generated by subcloning the 5′ UTR of L1 from the pYX014 plasmid into the pGluc-Basic plasmid (New England BioLabs, Ipswich, MA, USA). The reporter plasmid for L1 antisense promoter activity, pGLuc-ASP, was generated by subcloning the region between the *PstI* and *NheI* sites of the 5′ UTR of L1, as previously reported [29], in antisense orientation into the pGluc-Basic plasmid. For a plasmid expressing shRNA against TRPV1 (sh-TRPV1), a pair of oligos (5′-ACC GGG CTC AGA ATA ATT GCT AGG ATG TTA ATA TTC ATA GCA TCC TGG CAG TTA TTC TGA GCT TTT-3′ and 5′-CGA AAA AAG CTCA GAA TAA CTG CCA GGA TGC TAT GAA TAT TAA CAT CCT AGC AAT TAT TCT GAG CC-3′) were annealed and inserted into the *Bbs*I sites of pRSI9-U6-(sh)-UbiC-TagRFP-2A-Puro (Addgene #28289).

### 4.4. In Vitro RT Assay

The activity of the M-MLV RT (400 U/mL), SuperScript II Reverse Transcriptase (Thermo Fisher Scientific, Waltham, MA, USA), in the presence of a test compound (300 µM) was determined using the EnzChek Reverse Transcriptase Assay Kit (Thermo Fisher Scientific, Waltham, MA, USA) according to the manufacturer’s instruction. The values of treated wells were normalized with those of the control wells. 

### 4.5. L1 Retrotransposition Assay

The 293T cells were transfected with pYX014 using Lipofectamine 2000 reagent (Invitrogen, Waltham, MA, USA). At 24 h after transfection, cells were treated with the indicated concentrations of test compounds. After 3 days of treatment, *Firefly* and *Renilla* luciferase activities were measured using the Dual-Luciferase Reporter Assay System (Promega, Fitchburg, MA, USA) according to the manufacturer’s instruction in a single-well luminometer (Berthold, Lumat LB 9507, Bad Wildbad, Germany). Since *Renilla* luciferase was constitutively expressed from the reporter construct, *Firefly* luciferase activity was normalized to the corresponding *Renilla* luciferase activity. For knockdown experiments, 293T cells were co-transfected with an L1 reporter plasmid and a plasmid expressing sh-TRPV1. The assay was conducted in duplicate.

### 4.6. Cell Viability Assay

The 293T cells were treated with the indicated concentrations of test compounds for 3 days. The cell viability was determined using the CellTiter-Glo Luminescent Cell Viability Assay (Promega) according to the manufacturer’s instruction. Values of the treated wells were normalized to those of the control wells. The assay was conducted in duplicate.

### 4.7. L1 Promoter and ASP Assays 

The 293T cells were co-transfected with pGLuc-5′-UTR or pGLuc-ASP, together with pCMV-CLuc (New England BioLabs, Ipswich, MA, USA) using Lipofectamine 2000 reagent. At 24 h after transfection, cells were treated with the indicated concentrations of test compounds. After 3 days of treatment, the *Gaussia* and *Cypridina* luciferase activities were measured using the *Gaussia* and *Cypridina* Luciferase Assay Kit (New England BioLabs) according to the manufacturer’s instructions. *Gaussia* luciferase activity was normalized to the corresponding *Cypridina* luciferase activity.

### 4.8. Real-Time RT-PCR

Total RNA was extracted from the indicated cells and reverse transcribed using a Verso cDNA Synthesis Kit (Thermo Fisher Scientific) according to the manufacturer’s instruction. Quantitative real-time RT-PCR assays were carried out using Fast SYBR Green Master Mix (Thermo Fisher Scientific) and gene-specific primers with a QuantStudio 6 (Thermo Fisher Scientific). The assay was conducted in duplicate.

### 4.9. Primers

The sequences of the primers used in this study are as follows:

TRPV1-forward primer, 5′-ATG ACA GTG TGA TGG AGA GTC-3′

TRPV1-reverse primer, 5′-CCT TGC TGG ATC CTC TGT GG-3′

GAPDH-forward primer, 5′-AGC GAG ATC CCT CCA AAA TC-3′

GAPDH-reverse primer, 5′-AAA TGA GCC CCA GCC TTC TC-3′

### 4.10. Statistics

Statistical significance was assessed using a two-tailed Student’s *t*-test with a threshold of *p* < 0.05. 

## Supplementary Materials

The following are available online at http://www.mdpi.com/1422-0067/19/10/3243/s1, Table S1: Compounds derived from natural medicines screened in this study.
