# Supplementary material for: Inhibition of LINE-1 Retrotransposition by Capsaicin
Source: Int J Mol Sci. 2018 Oct 19;19(10):3243. doi: 10.3390/ijms19103243 (PMC6214084; doi:10.3390/ijms19103243)
Supplement: Supplementary file 1 [file ijms-19-03243-s001.pdf]

Supplemental Table S1. Compounds derived from natural medicines screened in this study

Aconitine	Dehydrocorydaline Nitrate	(Z)-Ligustilide
Albiflorin	Dehydrocostuslactone	Limonin
Alisol A	demethoxycurcumine	Liquiritin
Alisol B	Dihydrocapsaicin	Loganin
Alkannin	Dimethylesculetin	Luteolin
Amygdalin	Eleutheroside B	Magnolol
Arbutin	(-)-Epigallocatechin Gallate	Mesaconitine
Astragaloside IV	Epihesperidin	Naringin
Atractylenolide III	Ergosterol	Nodakenin
Atractylodin	beta-Eudesmol	Osthole
Atropine Sulfate	Evodiamine	Oxymatrine
Aucubin	(E)-Ferulic Acid	Paeoniflorin
Baicalein	Geniposide	Paeonol
Baicalin	Geniposidic Acid	Palmatine Chloride
Barbaloin	Gentiopicroside	Perillaldehyde
Benzoylmesaconine Hydrochloride	[6]-Gingerol	Praeruptorin A
Berberine Chloride	Ginsenoside-Rb1	Puerarin
Bergenin	Ginsenoside-Rc	Rhynchophylline
Bisdemethoxycurcumin	Ginsenoside-Rd	Rosmarinic Acid
Bufalin	Ginsenoside-Re	Saikosaponin a
Bufotalin	Ginsenoside-Rg1	Saikosaponin b2
Capillarisin	Glabridin	Saikosaponin c
(E)-Capsaicin	Glycyrrhizic Acid	Saikosaponin d
Catalpol	Gomisin A	Schizandrin
(E)-Chlorogenic Acid	Gomisin N	Sennoside A
(E)-Cinnamic Acid	Hesperidin	Sennoside B
Cinobufagin	Hirsutine	Shikonin
Cinobufotalin	Honokiol	[6]-Shogaol
Coptisine Chloride	Hypaconitine	Sinomenine
Corydaline	Icariin	Swertiamarin
Costunolide	Isofraxidine	Timosaponin A-Ⅲ
Curcumin	Isorhynchophylline	Wogonin
